# Orally administered boldine reduces muscle atrophy and promotes neuromuscular recovery in a rodent model of delayed nerve repair

**DOI:** 10.3389/fncel.2023.1240916

**Published:** 2023-09-27

**Authors:** Justin C. Burrell, Phuong T. Vu, Owen J. B. Alcott, Carlos A. Toro, Christopher Cardozo, D. Kacy Cullen

**Affiliations:** ^1^Department of Neurosurgery, Center for Brain Injury and Repair, Perelman School of Medicine, University of Pennsylvania, Philadelphia, PA, United States; ^2^Center for Neurotrauma, Neurodegeneration and Restoration, CMC VA Medical Center, Philadelphia, PA, United States; ^3^Department of Bioengineering, University of Pennsylvania, Philadelphia, PA, United States; ^4^Department of Biochemistry, Widener University, Philadelphia, PA, United States; ^5^Spinal Cord Damage Research Center, James J. Peters VA Medical Center, Bronx, NY, United States; ^6^Icahn School of Medicine, Mount Sinai, New York, NY, United States

**Keywords:** nerve regeneration, neuromuscular injury, muscle preservation, connexin hemichannels, delayed nerve repair

## Abstract

Peripheral nerve injury often results in poor functional recovery due to a prolonged period of muscle denervation. In particular, absent axonal contact, denervated muscle can undergo irrevocable atrophy and diminished receptiveness for reinnervation over time, ultimately reducing the likelihood for meaningful neuromuscular recovery. While innovative surgical approaches can minimize the harmful effects of denervation by re-routing neighboring—otherwise uninjured—axons, there are no clinically-available approaches to preserve the reinnervation capacity of denervated muscles. Blocking intramuscular connexin hemichannel formation has been reported to improve muscle innervation *in vitro* and prevent atrophy *in vivo*. Therefore, the current study investigated the effects of orally administered boldine, a connexin hemichannel inhibitor, on denervated-related muscle changes and nerve regeneration in a rat model of delayed peripheral nerve repair. We found that daily boldine administration significantly enhanced an evoked response in the tibialis anterior muscle at 2 weeks after common peroneal nerve transection, and decreased intramuscular connexin 43 and 45 expression, intraneural Schwann cell expression of connexin 43, and muscle fiber atrophy up to 4 weeks post transection. Additional animals underwent a cross nerve repair procedure (tibial to common peroneal neurorrhaphy) at 4 weeks following the initial transection injury. Here, we found elevated nerve electrophysiological activity and greater muscle fiber maturation at 6 weeks post repair in boldine treated animals. These findings suggest that boldine may be a promising pharmacological approach to minimize the deleterious effects of prolonged denervation and, with further optimization, may improve levels of functional recovery following nerve repair.

## Highlights

- Orally administered boldine reduced denervation-related muscle atrophy and loss of evoked electrical activity, and promoted neuromuscular recovery in a rat model of peripheral nerve injury.
- Boldine administration reduced intramuscular connexin 43/45 expression and muscle fiber atrophy, as well as connexin 43 expression around denervated Schwann cells up to 4 weeks post injury.
- Reinnervated muscle fiber diameters were larger in boldine-treated animals suggesting enhanced myofiber recovery.
- Intramuscular connexin 43/45 expression returned to baseline following delayed repair after stopping boldine treatment, indicating the treatment was reversible allowing for the continuation of physiological regenerative processes.

## Introduction

Peripheral nerve injury (PNI) routinely presents in approximately 3–5% of all trauma cases and may result in significant sensorimotor deficits and diminished quality of life (Robinson, 2000; Evans, 2001). Unfortunately, only approximately 50% of patients report satisfactory functional recovery after PNI, despite state-of-the-art surgical intervention (Ruijs et al., 2005; Pfister et al., 2011). Severe nerve injuries—most often associated with poor recovery—are denoted by axonal disconnection within the nerve sheath, effectively severing communication between the neuronal cell body (residing within or adjacent to the spinal cord) and its distal end target (e.g., muscle/sensory end organ) (Pfister et al., 2011). Such injuries initiate a series of choreographed degenerative and pro-regenerative processes; for instance, distal axon segments undergo Wallerian degeneration, denervated muscle upregulates acetylcholine receptor expression, and denervated Schwann cells form the bands of Bungner to create a pro-regenerative environment that facilitates axonal regeneration and muscle reinnervation (Fu and Gordon, 1995a,b). In particular, axonal degeneration results the loss of acetylcholine release followed by nicotinic acetylcholine receptor (AChR)-mediated muscle depolarization and muscarinic AChR-mediated activation of terminal Schwann cells, which initially serve to guide regenerating motor axons to neuromuscular junctions (Sugiura and Lin, 2011; Ko and Robitaille, 2015; Arbour et al., 2017). However, extended periods without axonal contact leads to the gradual degradation of this pro-regenerative environment and irrevocable muscle atrophy (Fu and Gordon, 1995a,b). Indeed, denervation impairs muscle membrane organization and reduces the stability of the neuromuscular junction by increasing nAChR degradation over nAChR turnover (Strack et al., 2015). Also, the regenerative capacity of initially pro-regenerative Schwann cells fades over time, resulting in dissolution of the bands of Bungner and ultimately Schwann cell apoptosis (Gomez-Sanchez et al., 2017). Therefore, successful reinnervation requires re-integration of the regenerating axons and denervated target muscle before the loss of the regenerative pathway and irrevocable atrophy minimize the likelihood of meaningful recovery. Thus, there is a significant clinical need to develop strategies to extend the optimal window for reinnervation.

Denervated muscle fibers remain viable after injury but undergo extensive atrophy, which is associated with *de novo* expression of connexin (Cx) hemichannels (HC) 43 and 45 within the cytoplasmic membrane of skeletal muscle fibers (sarcolemma) (Cea et al., 2013). Cx HC are non-selective membrane protein channels that most often couple with HC on adjacent cells to form gap junctions thereby directly connecting the cytoplasm of adjacent cells, which enables bidirectional transfer of ions or small molecules. In the sarcolemma, Cx HC cause physiological changes, such as increased permeability of the sarcolemma to calcium, ATP and other small molecules, reduced resting membrane potential and activation of muscle atrophy programs (Cea et al., 2013; Cisterna et al., 2016). Recently, Cx HC formation in denervated muscle has been reported to inhibit axon growth and muscle integration *in vitro* (Cisterna et al., 2020). Based on these findings, inhibiting Cx HC formation may be a potential therapeutic target following PNI. Previous studies have reported that Cx HC formation can be inhibited following boldine administration (Hernandez-Salinas et al., 2013; Yi et al., 2017; Cea et al., 2020). Therefore, the objective of this study was to assess the efficacy of orally administered boldine on muscle preservation, axonal regeneration, and functional recovery in a rat model of delayed nerve repair.

## Methods

### Animals

Male Sprague–Dawley rats (Charles River Laboratories; 300–330 g; aged 6–8 weeks) were used for all experiments. All procedures were approved by the Institutional Animal Care and Use Committees at the University of Pennsylvania and the Michael J. Crescenz Veterans Affairs Medical Center and adhered to the guidelines set forth in the NIH Public Health Service Policy on Humane Care and Use of Laboratory Animals (2015).

### Boldine administration

In this study, boldine was procured from a single vendor (Sigma B3916). Starting 7 days before surgery, rats were trained to eat peanut butter, a previously established oral vehicle for boldine (Cea et al., 2020). Rats were randomly assigned to the following groups: (A) peanut butter alone (vehicle control; *n* = 12 animals); (B) 50 mg/kg boldine in peanut butter (low dose, *n* = 12); or (C) 100 mg/kg boldine in peanut butter (high dose, *n* = 12). These doses were selected based on previous efficacy studies testing boldine in rats by our group and others (Jang et al., 2000; Hernandez-Salinas et al., 2013; Gomez and Velarde, 2018; Subramaniam et al., 2019; Toro et al., 2023). A technician fed rats beginning approximately 45 min after surgery and once daily thereafter. Animals enrolled in the denervated cohort (described below) received boldine once a day up to the terminal time point (4 weeks) whereas the delayed repair cohort (described below) was given boldine once a day up to 8 weeks post transection (4 weeks post delayed repair).

### Chronic rodent common peroneal nerve axotomy and prolonged denervation model

**Figure 1:**
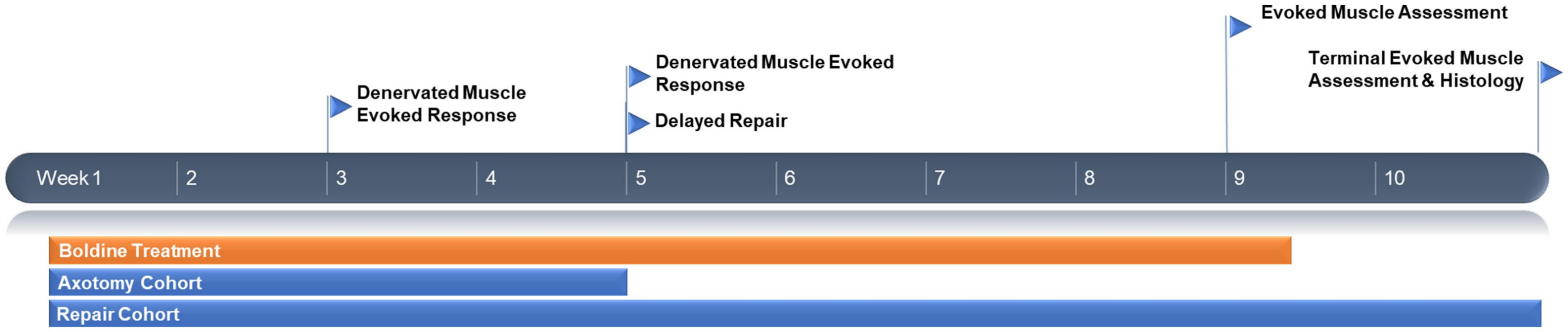
Experimental timeline.

To assess the effect of orally administered boldine on denervated muscle, we adapted a previously established rodent chronic axotomy model (Burrell et al., 2022). Briefly, animals were anesthetized with isoflurane and the hind leg cleaned with betadine. Meloxicam (2 mg/kg) was administered subcutaneously in the scruff of the neck and bupivacaine (2 mg/kg) was administered subcutaneously along the incision. The gluteal muscle was separated to expose the sciatic nerve exiting the sciatic notch. The common peroneal nerve was sharply transected and a 5 mm segment was removed, and the proximal stump was inserted in a nearby muscle to prevent axon regeneration and neuroma formation. The surgical site was closed with 4–0 absorbable vicryl sutures and skin staples. Animals were recovered and returned to the vivarium until the terminal time point of 4 weeks post nerve transection, with the administration of boldine (or vehicle) occurring daily over that entire time period (*n* = 6 animals for each group: control, low dose boldine, and high dose boldine). See Figure 1 for experimental timeline.

### Delayed cross-suture repair

A separate cohort of animals received a delayed nerve repair at 4 weeks following injury. Briefly, the animals were re-anesthetized and the surgical site was re-exposed. The denervated common peroneal nerve was identified as well as the previously uninjured tibial nerve. Delayed surgical repair was completed by sharply transecting the tibial nerve and refreshing the end of the previously denervated common peroneal nerve by removing approximately 5 mm of fibrotic tissue at the distal stump. Then, a standard end-to-end neurorrhaphy was completed using two 8–0 prolene sutures, securing the proximal tibial nerve with the distal common peroneal nerve. The deep layers and skin were closed, and the area was dressed as described above. Animals were recovered and returned to the vivarium until the terminal time point of 6 weeks after the delayed neurorrhaphy procedure (which was 10 weeks after initial axotomy). The administration of boldine (low or high dose) or vehicle (*n* = 6 animals per group) occurred daily until 4 weeks post delayed neurorrhaphy (which was 8 weeks following initial transection).

### Electrophysiological assessment

At 2 and 4 weeks post axotomy, and 4 and 6 weeks post delayed neurorrhaphy (corresponding to 8 and 10 weeks post axotomy), evoked muscle response was compared across groups following percutaneous stimulation. Animals were re-anesthetized and a bipolar subdermal stimulating electrode was placed percutaneously superficial to the common peroneal nerve (Burrell et al., 2022). A monopolar subdermal recording electrode was placed over the muscle belly of the tibialis anterior and the reference electrode placed in its tendon. After determining the initial threshold for evoked muscle recordings, the supramaximal recording was obtained by slowly increasing the current to maximize the amplitude to double the threshold current or until the waveform plateaued, and then averaged over a train of 5 pulses (biphasic; amplitude: 0–5 mA; duration: 0.2 ms; frequency: 1 Hz; 100x gain; 10–10,000 Hz band pass and 60 Hz notch filters; Natus Viking EDX).

At the terminal timepoints of 4 weeks post axotomy and 6 weeks post delayed neurorrhaphy, the surgical site was exposed for direct nerve stimulation and compound muscle action potentials (CMAP) and compound nerve action potentials (CNAP) recordings. To evaluate muscle reinnervation, CMAPs were obtained by stimulating 5 mm proximal or distal to the delayed repair site using a handheld bipolar hook electrode (biphasic; amplitude: 0–4 mA; duration: 0.2 ms; frequency: 1 Hz; 1,000x gain; 10–10,000 Hz band pass and 60 Hz notch filters; Natus Viking EDX). Mean peak-to-baseline amplitude were recorded. To assess the electrical conduction across the repair site, CNAPs were recorded by stimulating the proximal stump with a bipolar hook electrode and recording with a bipolar hook electrode (biphasic; amplitude: 0–2 mA; duration: 0.2 ms; frequency: 1 Hz; 1,000x gain; 10–10,000 Hz band pass and 60 Hz notch filters; Natus Viking EDX). Mean peak-to-peak amplitude was recorded and conduction velocity was calculated by dividing the distance between the electrodes by the latency between stimulation and evoked response.

### Euthanasia and tissue collection

At the terminal time points, animals were euthanized using carbon dioxide. Nerves were extracted and post-fixed in formalin for 24 h at 4°C, and then rinsed in PBS for another 24 h. Muscles were extracted in paraformaldehyde for 24 h at 4°C and then cryoprotected in 20% sucrose.

### Immunohistochemistry

For histological assessment, cryoprotected tissue was embedded in optimal cutting media, frozen in dry ice/isopentane, sectioned, and mounted on glass slides for staining. Nerves were sectioned axially at a thickness of 14 μm, 5 mm distal to the injury site. Muscles were sectioned axially at a thickness of 14 μm, starting 100 μm from the proximal muscle belly.

For cross-sectional muscle histology, frozen tissues were washed with PBS three times to remove embedding media, followed by 1 h of blocking and permeabilization in 4% normal horse serum and 0.3% Triton X-100. The primary antibodies were diluted in the previously described blocking solution. Rabbit anti-connexin 43 (1,100, Abcam, ab11370) and rabbit anti-connexin 45 (1,100, Abcam, ab135474) were used to label Cx43 and Cx45, respectively, and anti-synaptophysin (1,500, Abcam, ab32127) to label presynaptic vesicles specifically in the reinnervated cohort. After incubation in primary antibodies overnight at 4°C, the tissues were washed with PBS three times and incubated for 2 h at room temperature with the corresponding fluorophore-conjugated secondary antibody (1,1,000, AlexaFluor, Invitrogen) along with AlexaFluor-488-conjugated phalloidin (1,400, Invitrogen, A12379) to label muscle actin and AlexaFluor-647-conjugated bungarotoxin to label postsynaptic receptors (1,250, Invitrogen, B35450). Hoechst was applied for 10 min, then the stained tissues were mounted with Fluoromount G (Southern Biotech, 0100–20) and cover slipped.

For cross-sectional histological analyses of the post-repair distal nerve, frozen sections were washed three times with PBS and blocked for 1 h using the same blocking solution as for muscle histology. The primary antibodies were diluted in 4% of normal horse serum in Optimax (Biogenex), then applied to the sections overnight at 4°C. Anti-connexin 43 (1:100, Abcam, ab11370) and anti-connexin 45 (1,100, Abcam, ab135474) were used to label Cx43 and Cx45, rabbit anti-S100β (1,500, Invitrogen, PA1-38585) to label Schwann cells, chicken anti-myelin basic protein (Encor, CPCA-MBP; 1:1500) to identify myelin, mouse SMI31 (1,1,000, Biolegend, 801,602) and SMI32 (1,1,000, Biolegend, 801,701) to label axons, and rabbit anti c-Jun (1,200, Cell Signaling Technologies, 9,165 L) as an injury marker. After three PBS washes, the sections were incubated in the appropriate fluorophore-conjugated secondary antibody (1,1,000; AlexaFluor, Invitrogen) for 1 h at room temperature. Followed by incubation in Hoechst for 10 min. The staining process ended with Fluoromount G mounting and coverslipping.

### Data acquisition and statistical analyses

Data was collected by a blinded researcher (unique identifier for all samples) and automatically analyzed when possible (Panzer et al., 2020; Burrell et al., 2022). Cx43 and Cx45 expression was quantified using the open-source FIJI software, by first measuring the integrated density and the area of the negative control section to calculate its mean integrated density as the background fluorescence measurement. For each sample, the integrated density and area were obtained, then the corrected fluorescence was obtained per Region of Interest (ROI) with the following formula: Corrected Total Fluorescence = Integrated Density – (ROI Area x Background Fluorescence from Negative Control Section). Corrected mean fluorescence was calculated as Corrected Total Fluorescence/ROI Area, averaged across three samples per animal, and compared across groups.

Measurements of muscle fiber area were performed in FIJI software based on phalloidin fluorescence images obtained from a Keyence BZ-X800 microscope. Images were gray-scaled, Gaussian blurred, and converted to binary. The watershed algorithm was applied to separate muscle fibers on the images. The “Analyze Particles” function was then used to obtain the cross-sectional areas of muscle fibers with a size threshold between 200 and 6,000 μm^2^. Particles outside this range were assumed to be artifact and were removed prior to analyses.

Axon counts in distal nerves post repair were manually quantified using the count function in NIS elements software from a 40,000 μm^2^ ROI at high magnification in representative z-stacks at maximum projection. The number of non-myelinated axons were counted on SMI31/32 channel and the number of myelinated axons were counted on SMI31/32 + MBP channels. Axon areas and g-ratios were quantified from confocal z-stack maximum projections of two regions of interest (ROIs) per sample and analyzed using FIJI software. For each myelinated axon, the inner axon area and the outer (myelinated) axon area were obtained, and the g-ratio was calculated using a modified formula (Kaiser et al., 2021):


$$g-\text{ratio}=\sqrt{\frac{\text{Inner}\;\text{axon}\;\text{area}}{\text{Outer}\;\left(\text{myelinated}\right)\;\text{axon}\;\text{area}}}$$

For the quantification of S100β + cells (Schwann cells) co-localized with Cx43 and c-Jun in distal nerves of the denervated cohort, three representative ROIs were used per animal. The image processing pipeline in NIS elements entails gray-scaling, filtering with a rolling ball background subtraction, fluorescence and size thresholding (0.5–20 μm), watershed segmentation to separate objects, then cleaning smaller particles and filling holes. The function “HAVING” was applied to the Hoechst and S100β layers to obtain the number of S100β + cells (Schwann cells). The “HAVING” function was also applied to the Hoechst + S100β and Cx43 or c-Jun binary layers to quantify the number of cells co-expressing S100β and Cx43 or c-Jun.

To quantify the total number of acetylcholine receptors (AChR) and percentage of reinnervated neuromuscular junctions, three muscle sections per animal were imaged at low magnification to identify regions of bungarotoxin+ (BGX+) clusters of AchR (10x air objective, 1,024 × 1,024, Keyence BZ-X800 Fluorescence Microscope). AChR was identified as a region with BGX signal adjacent to edges of muscle fibers as marked by phalloidin. Next, three regions of interest (ROI) were randomly selected and automatically acquired (2 × 2 region, 100x oil objective with a 2x digital zoom, 2048 × 2048, Nikon A1R Confocal Microscope) without the researcher visualizing the synaptophysin channel prior to acquisition. BGX + cells or the total number of AchR receptors were quantified from the low magnification image from each animal. Mature neuromuscular junctions (NMJs) were identified as BGX + cells co-localized with synaptophysin-puncta adjacent to phalloidin + muscle fibers. Mean percent mature NMJs was calculated by dividing the number of mature NMJs by the total number of BGX + receptors, averaged across replicates and by group.

One-way ANOVA was performed on the following outcome metrics followed by post-hoc Tukey’s pairwise test when significant differences were found. For evoked muscle response assessment, mean peak-to-baseline amplitude and area-under-the-curve (AUC) was compared at 2 and 4 weeks following nerve injury, and 4 and 6 weeks post repair. For nerve and muscle electrophysiological function assessment, mean CMAP amplitude and CNAP peak-to-peak amplitude and conduction velocity were compared at 6 weeks post repair. For nerve regeneration assessment, mean SMI31 expression and axon size, and myelinated axon count were compared. For Schwann cell morphology assessment, percent of S100β-positive Schwann cells co-labeled with c-Jun were compared. For muscle histology, mean levels of Cx43 and Cx45, mean AchR count, and percent mature NMJ were compared at 6 weeks post delayed nerve repair. To assess muscle fiber diameter, at least 10,000 fibers were measured per group, with approximately the same number per animal, and the cumulative frequency distribution was plotted with the non-linear Gaussian line-of-best-fit. Plots were compared using the two-sample Kolmogorov–Smirnov test to evaluate the agreement between distribution profiles. For all statistical tests, *p* < 0.05 was required for significance (GraphPad Prism, La Jolla CA, United States). Mean values presented as mean ± standard deviation unless otherwise noted.

## Results

### The effects of boldine treatment on chronically denervated muscle

**Figure 2:**
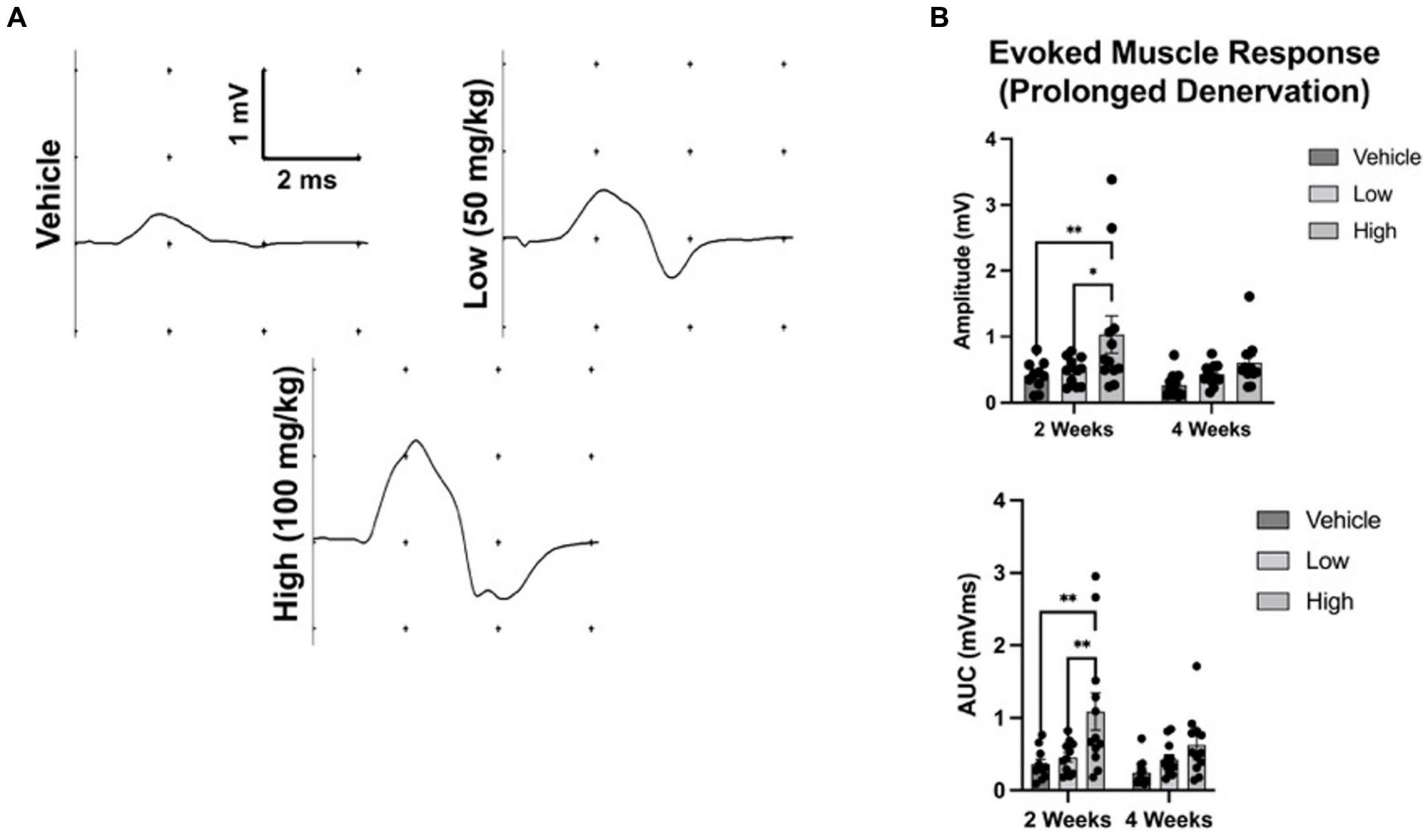
Oral administration of boldine preserves evoked muscle response following axotomy. To assess the effect of boldine administration on denervated muscle, rats were randomly enrolled into three groups: (a) vehicle; (b) low dose boldine (50 mg/kg); or (c) high dose boldine (100 mg/kg). In this model of prolonged denervation, the common peroneal nerve was transected and the proximal stump was sutured to a nearby muscle to prevent nerve regeneration. Animals were fed boldine in peanut butter or peanut butter alone (vehicle) every day starting immediately after nerve transection. Evoked muscle responses were assessed at 2 and 4 weeks following transection by placing recording electrodes over the muscle belly of the tibialis anterior (TA) muscle and stimulating electrodes over the denervated common peroneal nerve in close proximity to the TA muscle. **(A)** Representative waveforms at 2 weeks post injury are shown. Waveforms were recorded from every animal in this study. **(B)** At 2 weeks after injury, both the mean amplitude and area-under-the-curve (AUC) measurement of the evoked response were greater in the high and low dose boldine groups compared to the vehicle group. However, by 4 weeks, there were no statistically significant differences in amplitude or AUC across groups. These findings suggest boldine may preserve the evoked muscle response of a denervated muscle for up to 2 weeks post injury. Bars represent Mean Value ± Standard Deviation. *p < 0.05, **p < 0.01, *n* = 10–12 per group.

To assess whether boldine administration blocks Cx HC formation and preserves the evoked electrophysiological response, we utilized a well-established rodent model of chronic axotomy and prolonged muscle denervation (Fu and Gordon, 1995b; Burrell et al., 2022). At 2 weeks following denervation, the amplitude of the evoked muscle response was greater in the high dose boldine cohort compared to control groups (Figure 2; vehicle: 0.41 ± 0.22 mV, low: 0.48 ± 0.21 mV, high: 1.03 ± 0.98). However, no differences in mean amplitude were detected at 4 weeks post denervation (vehicle: 0.27 ± 0.19 mV, low: 0.43 ± 0.16 mV, high: 0.6 ± 0.36 mV). Similarly, the mean area-under-the-curve (AUC) at 2 weeks was greater in the high dose cohort (vehicle: 0.36 ± 0.22 mVms, low: 0.46 ± 0.22 mVms, high: 1.09 ± 0.9 mVms), and there was no significant differences at 4 weeks (vehicle: 0.24 ± 0.19 mVms, low: 0.43 ± 0.22 mVms, high: 0.63 ± 0.42 mVms).

**Figure 3:**
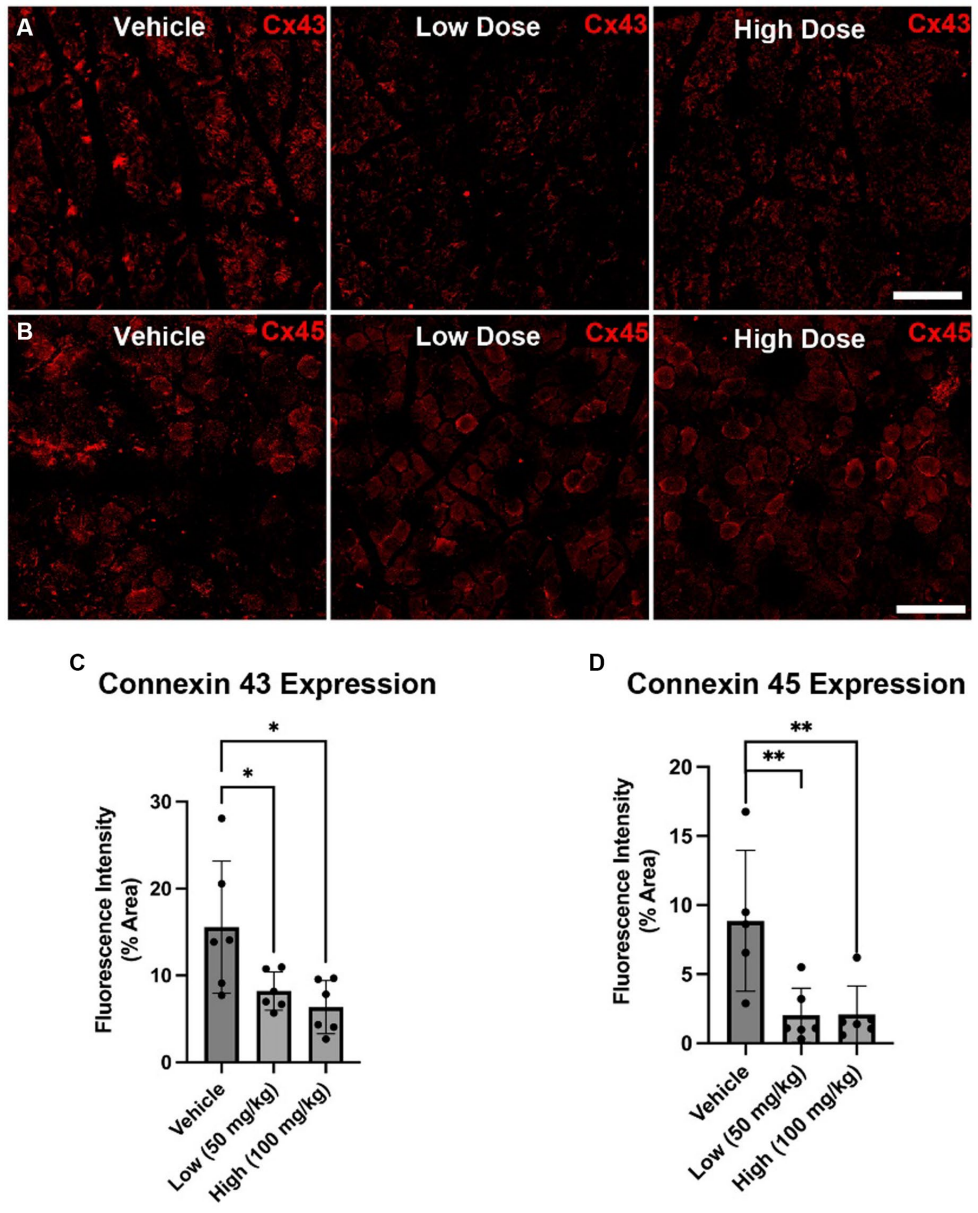
Cx43 and Cx45 expression in denervated muscle at 4 weeks following axotomy. Immunohistochemistry was performed on denervated TA muscle harvested at 4 weeks following common peroneal nerve transection. **(A,B)** Representative images are shown labeling for Cx43 **(A)** and Cx45 **(B)** Scale Bar: 150 μm. **(C,D)** At 4 weeks post injury, reduced Cx43 **(C)** and Cx45 **(D)** expression was found in the low dose cohort compared to the other cohorts. These findings suggest that boldine administration may decrease intramuscular Cx43 and Cx45 HC expression following axotomy. Bars represent Mean Value ± Standard Deviation. *p < 0.05, **p < 0.01, *n* = 5–6 per group.

**Figure 4:**
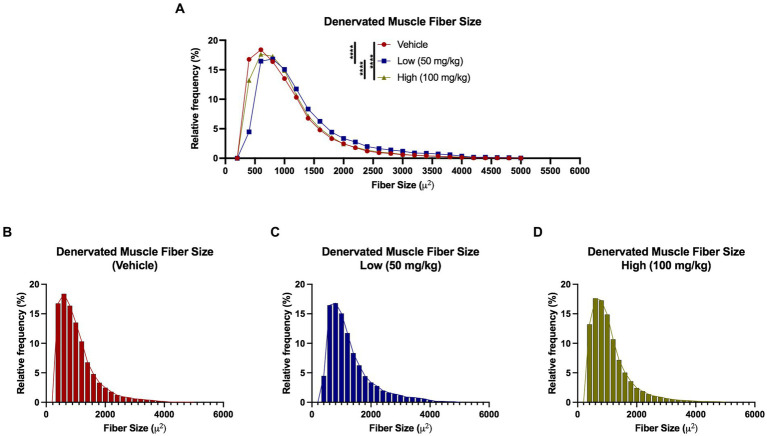
Denervated muscle fiber size histograms at 4 weeks following axotomy. Muscle fibers were automatically segmented from cross-sectional samples stained with phalloidin. **(A–D)** A rightward shift observed in both low and high dose groups compared to vehicle, indicating a greater percentage of denervated muscle fibers had a larger fiber area following boldine treatment. These findings suggest boldine may attenuate muscle fiber atrophy following denervation. Break out histograms from each group are provided.

**Figure 5:**
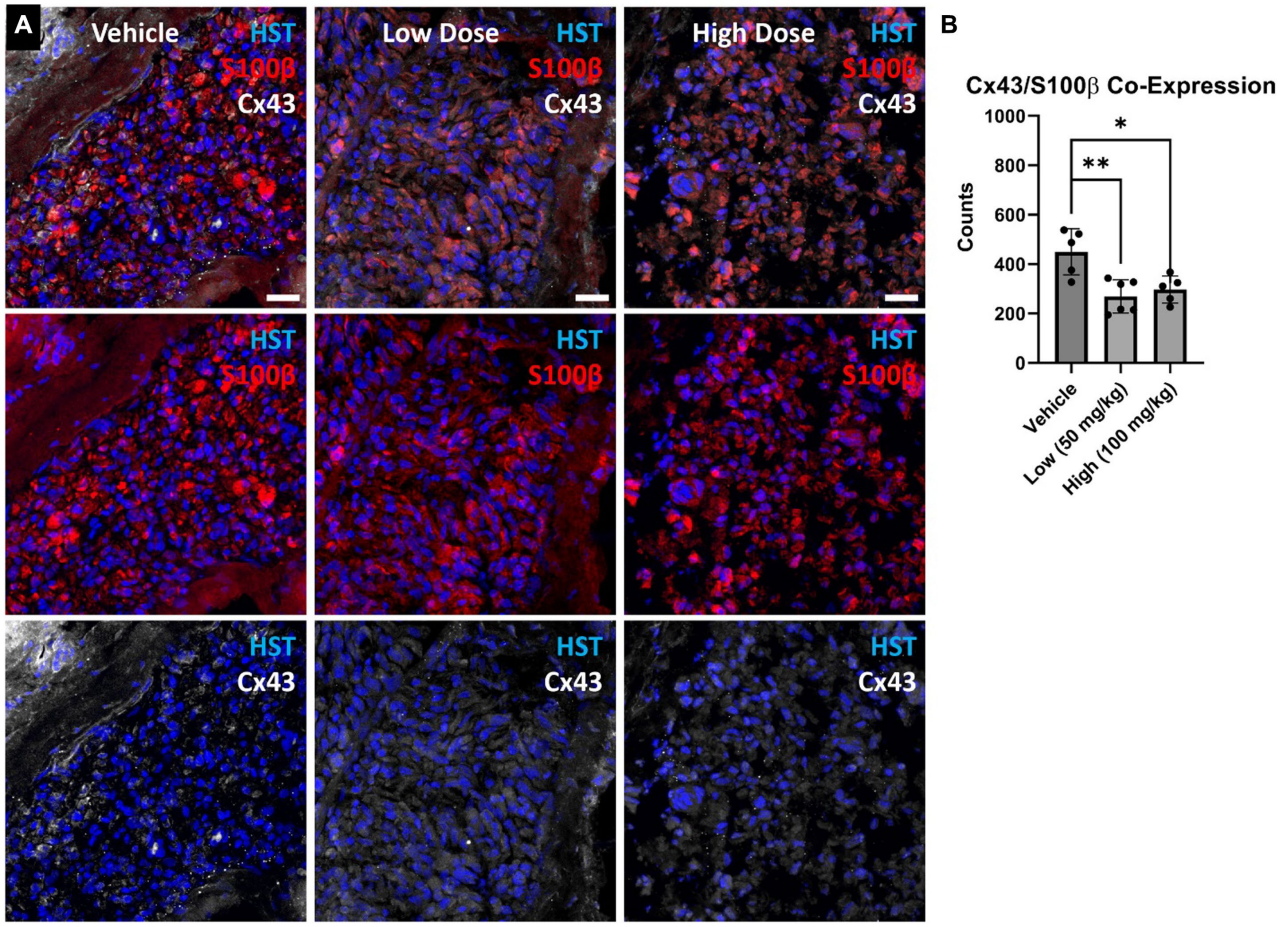
**(A)** Denervated cross-sectional nerve segments were stained for Schwann cells (S100β) and Connexin 43 (Cx43). **(B)** Fewer S100β+ cells co-expressing Cx43 were found in the boldine cohorts compared to vehicle. These findings suggest boldine may reduce connexin hemichannel formation along Schwann cells within the denervated nerve after injury. Scale Bar: 20 µm. Bars represent Mean Value ± Standard Deviation. *p < 0.05, **p < 0.01, *n* = 5–6 per group.

At 4 weeks post axotomy, the tibialis anterior muscle was isolated and weighed for all groups. While there was a trend toward increased mean muscle weight in the low dose cohort, there were no statistical differences found (Supplementary Figure S1). We also performed immunocytochemistry on muscle sections to measure levels of Cx43 and Cx45. Here, we found greater levels of Cx43 and Cx45 in the control group compared to the boldine-treated groups, indicating that boldine treatment successfully prevented Cx expression following prolonged denervation (Figure 3). Similarly, we measured the denervated muscle fiber area and found that both boldine groups had a rightward shift compared to the controls, suggesting attenuated fiber atrophy following boldine treatment (Figure 4, vehicle median fiber area: 882.97 μm^2^, low dose: 1062.06 μm^2^, high dose: 924.62 μm^2^). Indeed, 25% of the fibers measured in the vehicle group had an area less than 587.25 μm^2^, compared to 25% of the fibers in the low and high cohort had an area less than 749.68 μm^2^ and 637.23 μm^2^, respectively. Approximately 25% of the fibers measured in the vehicle group had an area greater than 1,000 μm^2^, compared to 35.4 and 26.3% of the fibers in the low and high cohort, respectively. Interestingly, the low dose group was significantly different from the high dose group. While the main goal of this study was to assess the effects of boldine on prolonged muscle denervation, previous studies have shown that Cx43 may play a role in nerve development, injury, and regeneration processes, specifically in the perineurium and Schwann cells (Chandross et al., 1996; Yoshimura et al., 1996). Indeed, we found fewer Schwann cells (labeled with S100β) co-expressing Cx43 at 4 weeks after injury in the high dose cohort compared to the other groups (Figure 5; vehicle: 449.7 ± 93.56 cells/mm^2^, 269.1 ± 67.18 cells/mm^2^, 297.4 ± 55.06 cells/mm^2^). Notably, no differences were found in the number of nuclei expressing pro-regenerative transcription factor c-Jun within the denervated nerve (vehicle: 196.0 ± 32.95 cells/30,000 μm^2^ low dose: 176.0 ± 57.27 cells/30,000 μm^2^, high dose: 145.1 ± 51.94 cells/30,000 μm^2^) or the number of c-Jun-positive nuclei within S100β-positive Schwann cells (Supplementary Figure S2; vehicle: 143.5 ± 44.56 cells/30,000 μm^2^ low dose: 145.1 ± 48.57 cells/30,000 μm^2^, high dose: 125.9 ± 46.38 cells/30,000 μm^2^).

### The effects of boldine treatment on neuromuscular recovery after delayed nerve repair

To assess whether boldine administration promotes functional recovery, a delayed neurorrhaphy was performed at 4 weeks post transection in a manner similar to those described in previous studies (Fu and Gordon, 1995b; Burrell et al., 2022).

**Figure 6:**
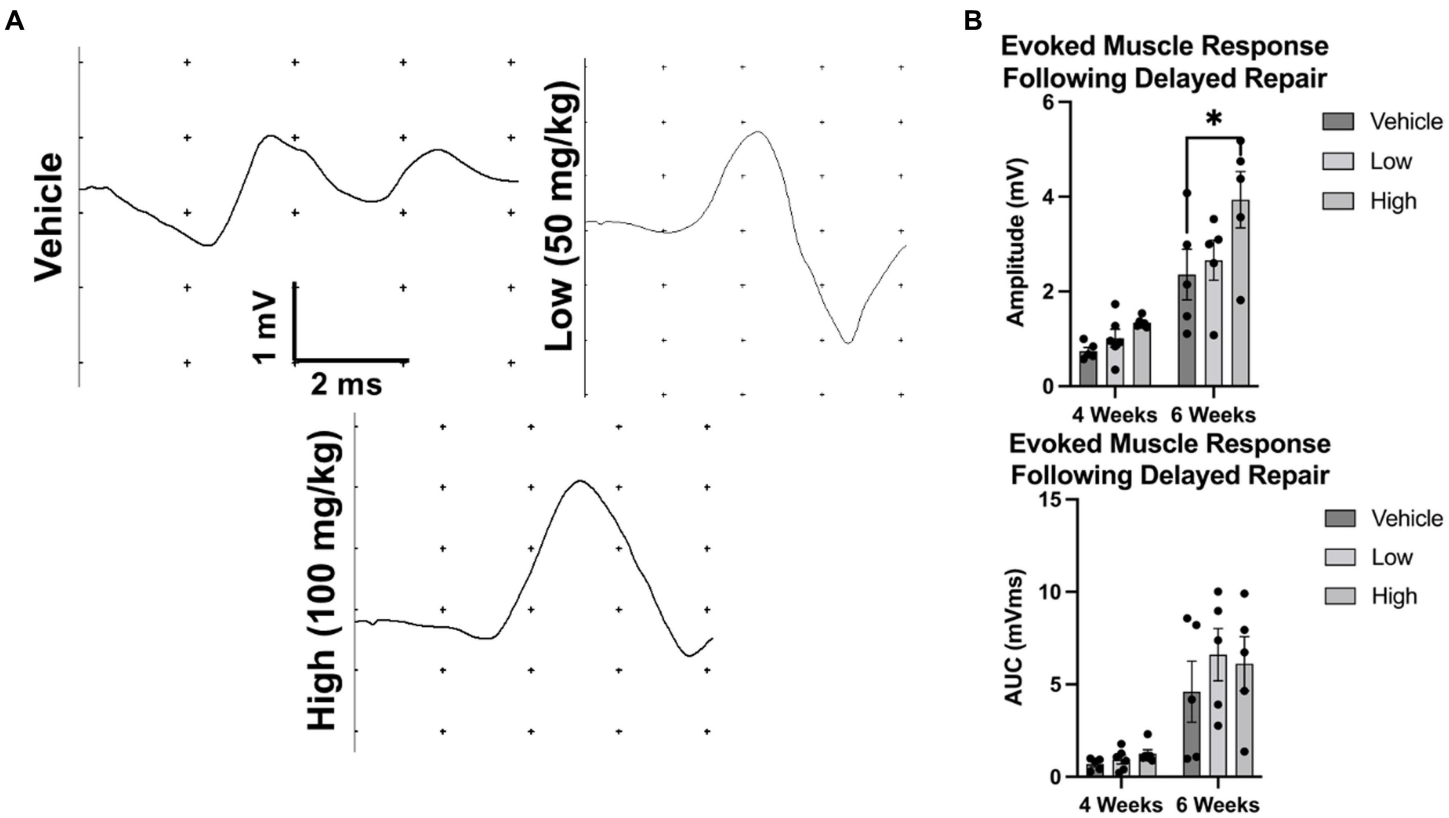
Evoked muscle response at 4 and 6 weeks following delayed repair. To assess electrophysiological function after delayed nerve repair, rats were randomly enrolled into the vehicle, low dose, or high dose groups. The common peroneal nerve was transected and animals were fed peanut butter daily (with or without boldine, depending on group). **(A)** Representative evoked muscle responses are shown following percutaneous stimulation. **(B)** No differences in the amplitude of the evoked muscle response were observed at 4 weeks post repair or the mean AUC at 4 or 6 weeks post repair; however, mean amplitude of the evoked muscle response was greater in the high dose cohort than vehicle at 6 weeks post repair. Bars represent Mean Value ± Standard Deviation. *p < 0.05, **p < 0.01, *n* = 5–6 per group.

Evoked muscle responses were measured at 4 and 6 weeks post repair. At 4 weeks post repair, the evoked muscle response was similar between groups; however, by 6 weeks post repair, a greater response was detected in the high dose cohort compared to vehicle (vehicle: 2.36 ± 1.2 mV, low: 2.66 ± 0.95 mV, high: 3.94 ± 1.33 mV) (Figure 6). No statistical differences were found for mean AUC at 4 or 6 weeks post delayed repair. At the terminal time point (6 weeks post delayed repair), intraoperative muscle and nerve electrophysiology was assessed. Compound muscle action potentials were comparable across treatment groups (Supplementary Figure S4). Greater compound nerve action potential magnitudes were found in the low dose group compared to the vehicle control group: (Supplementary Figure S3; 226.9 ± 187.8 μV, low: 640.6 ± 290.4 μV, high: 409 ± 222.7 μV); however, conduction velocities were equivalent between groups (vehicle: 32.54 ± 9.12 m/s, low: 36.43 ± 7.16 m/s, high: 38.96 ± 13.57 m/s).

**Figure 7:**
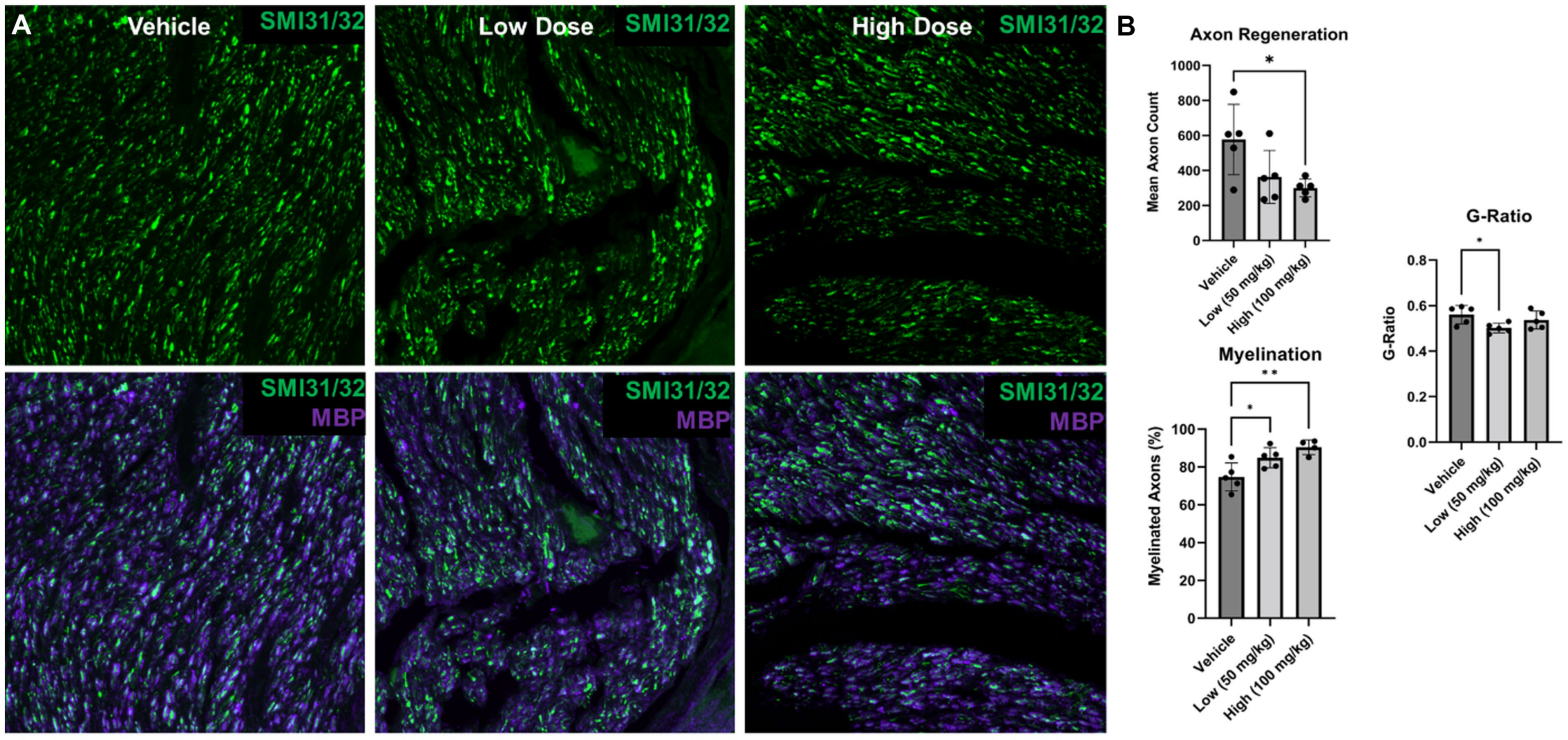
Nerve morphometry at 6 weeks following delayed repair. Immunohistochemistry was performed on distal common peroneal nerve at 6 weeks following delayed nerve repair. (A) Representative images are shown labeling regenerating axons (SMI31/32; green) and myelin basic protein (MBP; purple) for vehicle, low and high dose, respectively. **(B)** At 6 weeks post delayed repair, fewer regenerated axons were found in the high dose boldine group compared to the vehicle control; however, greater myelination was observed in the low and high dose boldine experimental groups compared to the vehicle group. Significant differences in g-ratio were also found between the low dose boldine compared to the vehicle group. Scale Bar: 20 μm. Bars represent Mean Value ± Standard Deviation. *p < 0.05, **p < 0.01, *n* = 5–6 per group.

To further contextualize the electrophysiological recovery findings, nerve regeneration and myelination was assessed at 6 weeks post delayed repair (Figure 7). Nerve regeneration was compared by quantifying the axon density distal to the repair site. At 6 weeks post repair, similar regeneration was detected between the vehicle group and low dose boldine group; however, the axon density was reduced in high dose group compared to the vehicle group (vehicle: 577.0 ± 89.80 axons/30,000 μm^2^, low: 363.6 ± 67.63 axons/30,000 μm^2^, high: 300.2 ± 22.71 axons/30,000 μm^2^). Further analysis revealed greater myelination in the boldine treated groups compared to the vehicle control group (vehicle: 74.85 ± 3.30%, low: 84.9 ± 2.39%, high: 90.5 ± 1.93%), suggesting boldine administration may promote nerve maturation at this early timepoint.

**Figure 8:**
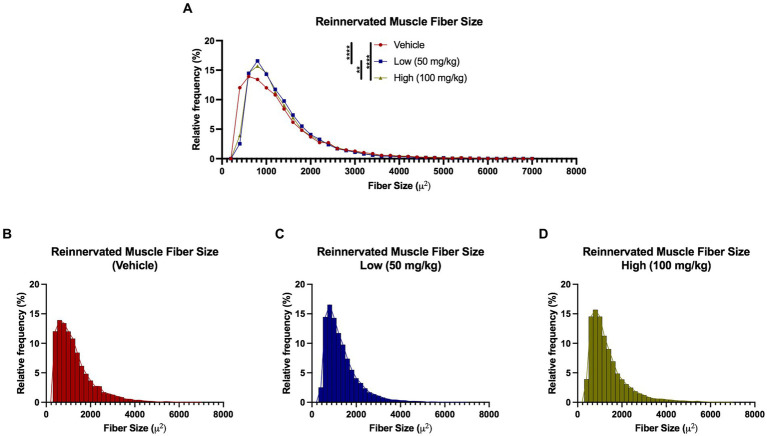
Reinnervated muscle fiber size histograms at 6 weeks following delayed repair. Muscle fibers were automatically segmented from cross-sectional samples stained with phalloidin. **(A–D)** Histograms were generated by measuring fibers per animal that were grouped by experimental treatment. A leftward shift was observed in the vehicle group compared to the boldine groups, suggesting diminished recovery from muscle fiber atrophy at 6 weeks post delayed repair in the vehicle-treated animals. Break out histograms from each group are provided.

Detailed muscle morphometric analyses in the reinnervation cohort revealed significant differences between the groups (Figure 8). A leftward shift was observed in the vehicle group compared to both of the boldine groups (vehicle median fiber area: 1078.72 μm^2^, low dose: 1137.03 μm^2^, high dose: 1120.36 μm^2^). Moreover, 25% of the fibers in the vehicle group were less than 687.21 μm^2^, compared to 25% of the fibers in the low and high cohort had an area less than 795.50 and 778.85 μm^2^, respectively. Approximately 60.3% of fibers were greater than 1,000 μm^2^ compared to 66.45 and 65.83% in the low dose and high dose groups, respectively. Additionally, no significant differences in Cx43 and Cx45 expression was found across groups at this time point (Supplementary Figure S5). Collectively, these findings suggest boldine administration may enhance muscle fiber recovery from atrophy without disrupting the intrinsic regeneration and maturation mechanisms.

**Figure 9:**
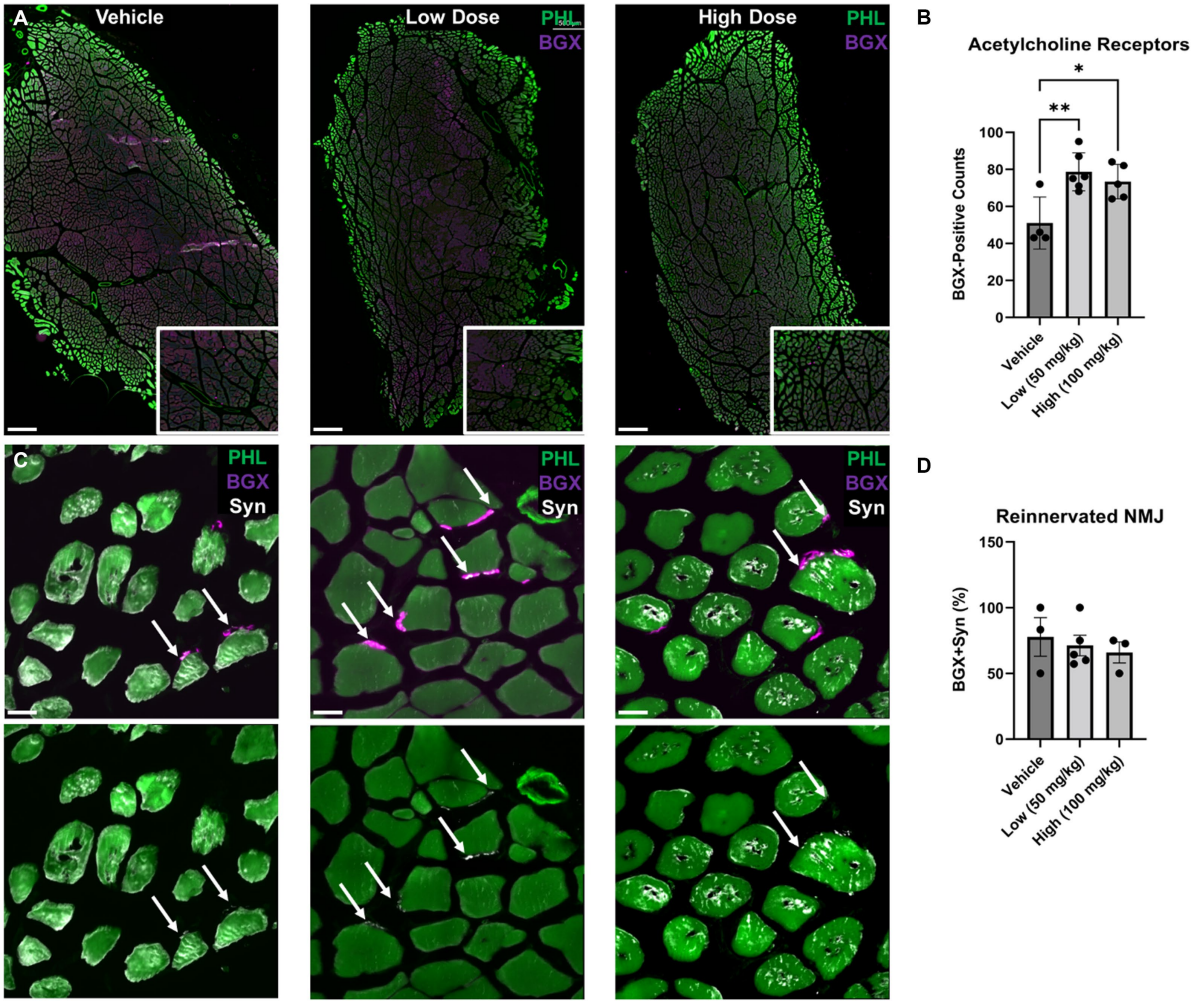
Acetylcholine receptor expression and neuromuscular junction formation at 6 weeks following delayed repair. Immunohistochemistry was performed on the TA muscle at 6 weeks following delayed nerve repair. **(A)** Representative images at low magnification are shown identifying bungarotoxin (BGX)-labeled acetylcholine receptors (AchR) in purple counterstained with phallodin (PHL)-labeled muscle fiber in green. Digital zoom-in insets display BGX expression surrounding PHL-positive muscle fibers. Scale bar: 500 μm. **(B)** At 6 weeks post delayed repair, greater AchR expression was observed in the low and high dose boldine cohorts compared the vehicle control group. **(C)** Representative images using high magnification, high resolution confocal microscopy revealed reinnervated neuromuscular junctions (NMJs) based on synaptophysin-(Syn) co-localization with BGX+ AchR. Scale bar: 20 μm. **(D)** No statistical differences were detected for reinnervated NMJs. Bars represent Mean Value ± Standard Deviation. *p < 0.05, **p < 0.01, *n* = 3–6 per group.

To determine the effect of boldine on neuromuscular junction reinnervation, muscle sections were stained with bungarotoxin (BGX) and synaptophysin (Syn) to label acetylcholine receptors and presynaptic vesicles, respectively (Figure 9). At 6 weeks post neurorrhaphy, significant elevation in BGX-labeled acetylcholine receptor expression was measured following boldine administration compared to the vehicle controls (vehicle: 51.0 ± 7.04, low: 78.7 ± 4.20, high: 73.4 ± 4.17). However, no statistical differences were detected in the percent co-localization of BGX and Syn (vehicle: 77.8 ± 14.70%, low: 71.3 ± 7.79%, high: 65.9 ± 7.98%). These findings indicate that boldine administration may preserve the capacity for muscle reinnervation by preventing acetylcholine receptor degradation at this early time point.

## Discussion

In this study, the efficacy of daily oral boldine administration was assessed in a rodent model of prolonged denervation and delayed nerve repair. Notably, boldine administration preserved the evoked muscle for 2 weeks after injury and mitigated muscle fiber atrophy up to 4 weeks following axotomy. Based on these findings, we hypothesized that boldine might promote functional recovery by preventing the harmful effects of prolonged denervation. Therefore, we next performed a delayed nerve repair to examine the effect of boldine administration on nerve regeneration, muscle reinnervation, and muscle electrophysiological recovery. Notably, greater axon myelination, reinnervated muscle fiber diameter, and acetylcholine receptor expression were measured at 6 weeks post repair (10 weeks total from initial transection injury). However, an important caveat to note is that it is difficult to de-couple the direct and indirect effects of boldine on regeneration. For instance, boldine may prevent muscle atrophy and the healthier muscle promotes axon maturation and reinnervation. Collectively, these findings suggest boldine may attenuate muscle atrophy and promote early maturation of regenerated axons; however, additional work is necessary to determine its effectiveness as an adjunctive pharmaceutical approach to mitigate prolonged denervation.

Seminal research on denervation in skeletal muscles established that when nerves are transected close the muscle, electrophysiological recovery is diminished at an earlier time point than in the case of a more proximal injury. A notable observation was that the longer the nerve stump, the longer the time it can transmit electrical signals to the muscle (Eyzaguirre et al., 1952; Birks et al., 1960). Cisterna et al. identified acetylcholine as the neuron-derived factor responsible for this phenomenon (Cisterna et al., 2020). The nerve stump stores and releases acetylcholine as a protective factor, which activates nicotinic acetylcholine receptors through a post-transcriptional process that suppresses Cx HC expression. Cx43 and Cx45 have been linked to adverse effects on the reinnervation process and blocking them slows the progression of muscle atrophy following denervation (Cea et al., 2020). The mechanism proposed by Cisterna et al. for myofiber alterations following denervation involves a reduction in acetylcholine release, which leads to the formation of Cx HC and an increase in intracellular Ca^2+^ concentration. This Ca^2+^ influx signals protein degradation in the denervated muscles. Therefore, Cx HC mediated calcium signaling initiates the denervated muscle atrophy process and hinders muscle reinnervation.

Boldine is a special type of Cx HC blocker in that it blocks movement of small molecules through the HC without preventing gap junction communication, which is essential for proper physiological function (Hernandez-Salinas et al., 2013; Yi et al., 2017). Additionally, boldine treatment has been reported decrease Cx43 and Cx45 expression in murine myofibers and successfully restored normal innervated myofiber phenotype (Cea et al., 2020). Moreover, as an acetylcholine esterase inhibitor, boldine prevents the degradation of acetylcholine, increasing its half-life and thus delaying the deleterious impact of denervation on myofibers (Kostelnik and Pohanka, 2018). Our results in the denervation cohort are consistent with previous findings about the role of Cx43/45 and the efficacy of boldine treatment. The control group, which had a higher degree of atrophy as measured by muscle mass and myofiber diameter, expressed higher Cx43/45 immunoreactivity than the group receiving low dose boldine. Similar to previous studies (Cea et al., 2012), we report denervated muscle fibers upregulate Cx43/45 HC after injury that can be ameliorated with boldine treatment. Notably, a recent study tested boldine administration (50 mg/kg) in mice after spinal cord injury and found treatment did not prevent body weight or muscle weight atrophy (Potter et al., 2023). In this study, we found that boldine administration preserved the evoked muscle response up to 2 weeks after injury, suggesting boldine may maintain the denervated muscle electrophysiological activity.

Previous work has shown Cx43/45 HC are important for muscle regeneration (Araya et al., 2005; Cea et al., 2012) and detrimental to reinnervation (Cisterna et al., 2020). However, the role of Cx43/45 in nerve regeneration remains unclear. Immediately after birth, Cx43 expression upregulates and then gradually decreases (Yoshimura et al., 1996); however, adult nerves express Cx43 in the perineurium (Chandross et al., 1996) and localized in Schwann cells body (Yoshimura et al., 1996). Intraneural Cx43 expression has also been reported to upregulate after injury and downregulate to baseline during regeneration, and may be important the myelination process (Chandross et al., 1996). Therefore, boldine administration was stopped at 4 weeks after delayed repair, which was calculated to be the approximate minimal time point for early reinnervation. At 6 weeks post delayed repair (2 weeks after boldine administration ended), intramuscular Cx43/45 HC expression was similar across groups; however, reinnervated muscle fiber diameter was greater in the boldine cohort. These results suggest that boldine can be used to prevent the harmful consequences of denervation without causing permanent damage to the intrinsic processes involved in muscle regeneration.

While connexin hemichannels are known to play an important role in the communication between myelinating Schwann cells and axons (Cisterna et al., 2019), to the best of our knowledge, this is the first study to assess the effects of boldine administration after nerve injury and following delayed nerve repair. Interestingly, we found that boldine treatment decreased Cx43 expression surrounding Schwann cells after nerve injury; however, additional experiments are necessary to elucidate any potential changes in pro-regenerative Schwann cell function. After delayed nerve repair, we found fewer regenerating axons in the distal nerve following boldine administration; however, there was enhanced myelination suggesting a more mature phenotype of regenerating axons. Additional studies are necessary to elucidate the mechanisms underlying these findings. Notably, the effect of boldine treatment on Cx43/45 may be reversible after terminating administration, allowing for normal maturation and myelination processes. Similarly, acetylcholinesterase has been reported to impede axonal extension and then promote outgrowth upon inhibition, and boldine may have similar paradoxical effects (Keymer et al., 1999). Multi-omic analyses using engineered test beds comprised of three dimensional neurons co-cultured with Schwann cells (and potentially myocytes) may be warranted to evaluate the effect of boldine following denervation and reinnervation in a well-controlled platform (Panzer et al., 2020). Overall, boldine treatment does not appear to impact regeneration at the early time points investigated in this study; however, future efficacy studies may be necessary to further evaluate the chronic effects on functional recovery.

After PNI, surgeons often utilize a wait-and-see approach prior to surgical intervention in hope of spontaneous recovery. While some patients may recover, more severe cases requiring delayed surgical reconstruction have a diminished likelihood for restoration. This study aimed to assess the efficacy of orally administered boldine as potential pharmaceutical intervention that promotes functional recovery after delayed nerve repair. Although oral administration of boldine has been previously reported (Cea et al., 2020), an important limitation of this study is that boldine has a short half-life. Therefore, future studies may include determining the optimal dose and timing, as well as the alternative formulations for local administration and/or sustained release after nerve injury. Prior to completing efficacy testing in a large animal model, a dosage study must be performed to establish a safe and effective dose range, rather than simply scaling the effective rodent dose by body weight. For example, a scaled safe and effective dose range can be calculated based on the species body weight and body surface area (Nair and Jacob, 2016). While boldine may be a promising intervention, additional preclinical testing in large animal models is necessary to determine the mechanism(s)-of-action and evaluate functional recovery (Burrell et al., 2020, 2021; Petrov et al., 2022). Additionally, pharmacological treatment of muscle denervation may be a useful adjunct for advanced tissue engineering strategies that replace damaged nervous structures (Zhang et al., 2018a,b, 2021, 2022; Katiyar et al., 2020, 2021; Shultz et al., 2021; Burrell et al., 2022; Smith et al., 2022). In summary, our findings demonstrate promising effects of boldine administration on preservation of muscle function following nerve injury as well as on enhancing neuromuscular functional recovery after delayed nerve repair.

## Data availability statement

The raw data supporting the conclusions of this article will be made available by the authors upon request, without undue reservation.

## Ethics statement

The animal study was approved by the Institutional Animal Care and Use Committees at the University of Pennsylvania and the Michael J. Crescenz Veterans Affairs Medical Center. The study was conducted in accordance with the local legislation and institutional requirements.

## Author contributions

JB: conceptualization, methodology, validation, investigation, data curation, visualization, writing—original draft, writing—review and editing, formal analysis, project administration, supervision. PV: methodology, validation, investigation, data curation, writing—original draft, writing—review and editing. OA: data curation. CT: conceptualization, writing—review and editing. CC: conceptualization, writing—review and editing, funding acquisition. DC: conceptualization, methodology, writing—review and editing, project administration, resources, supervision, funding acquisition. All authors contributed to the article and approved the submitted version.
