# Supplementary figures and images for: Orally administered boldine reduces muscle atrophy and promotes neuromuscular recovery in a rodent model of delayed nerve repair
Source: Front Cell Neurosci. 2023 Sep 27;17:1240916. doi: 10.3389/fncel.2023.1240916 (PMC10565860; doi:10.3389/fncel.2023.1240916)

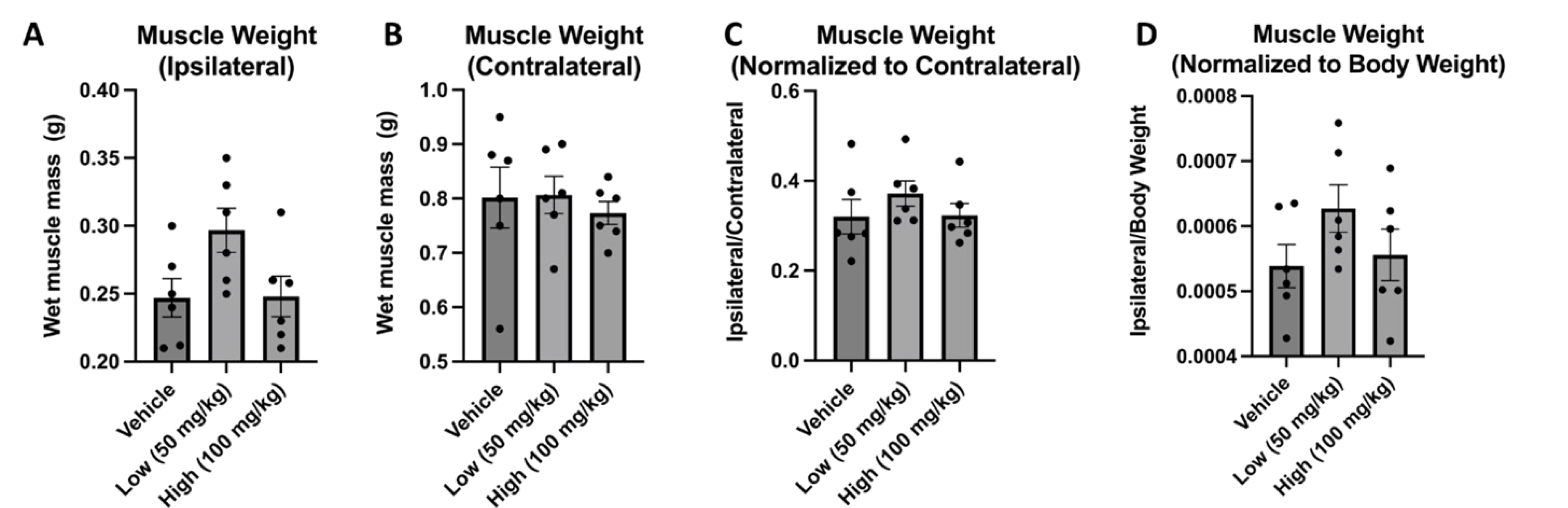

Supplement: Supplementary file 1 [file Image_1.tif]

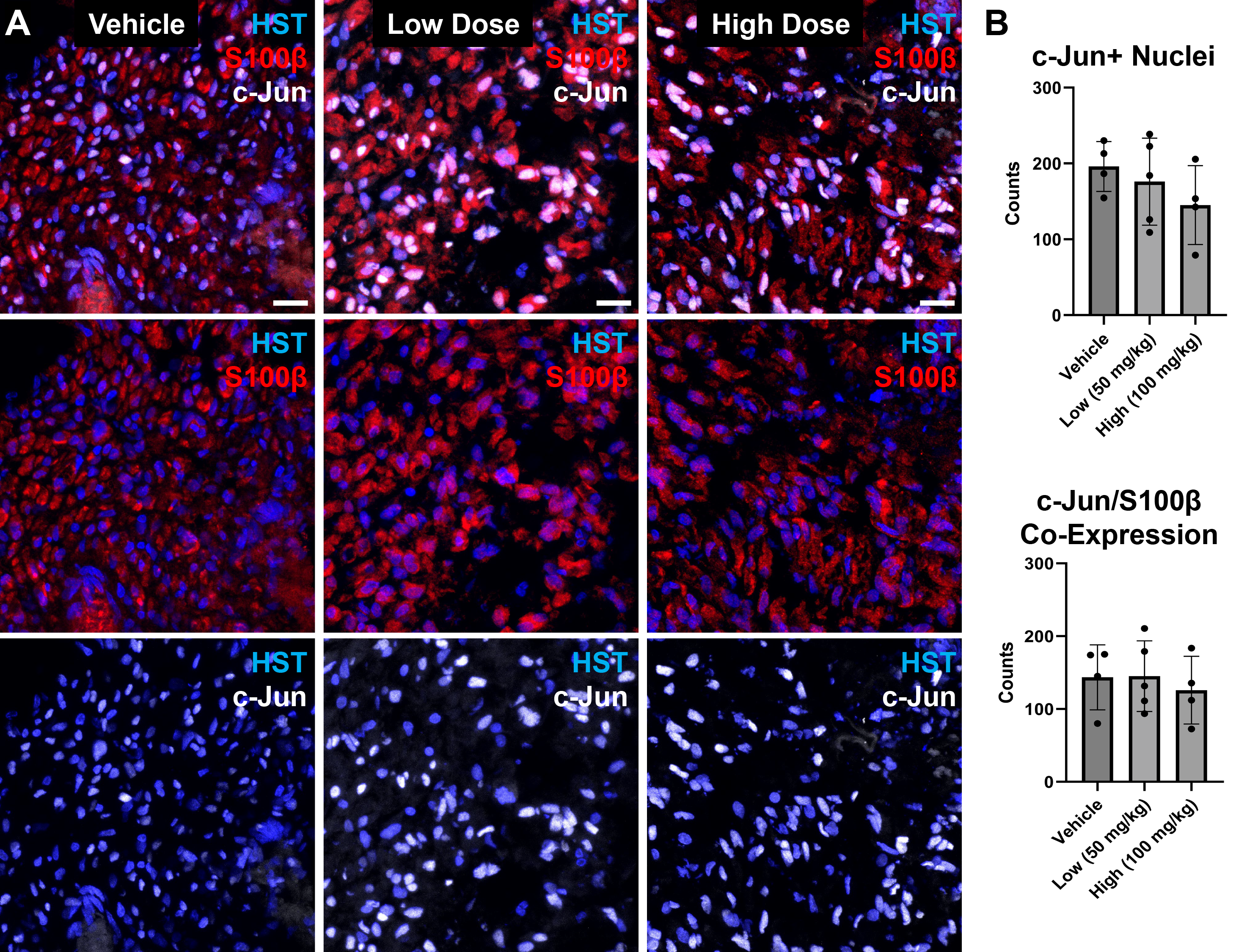

Supplement: Supplementary file 2 [file Image_2.tif]

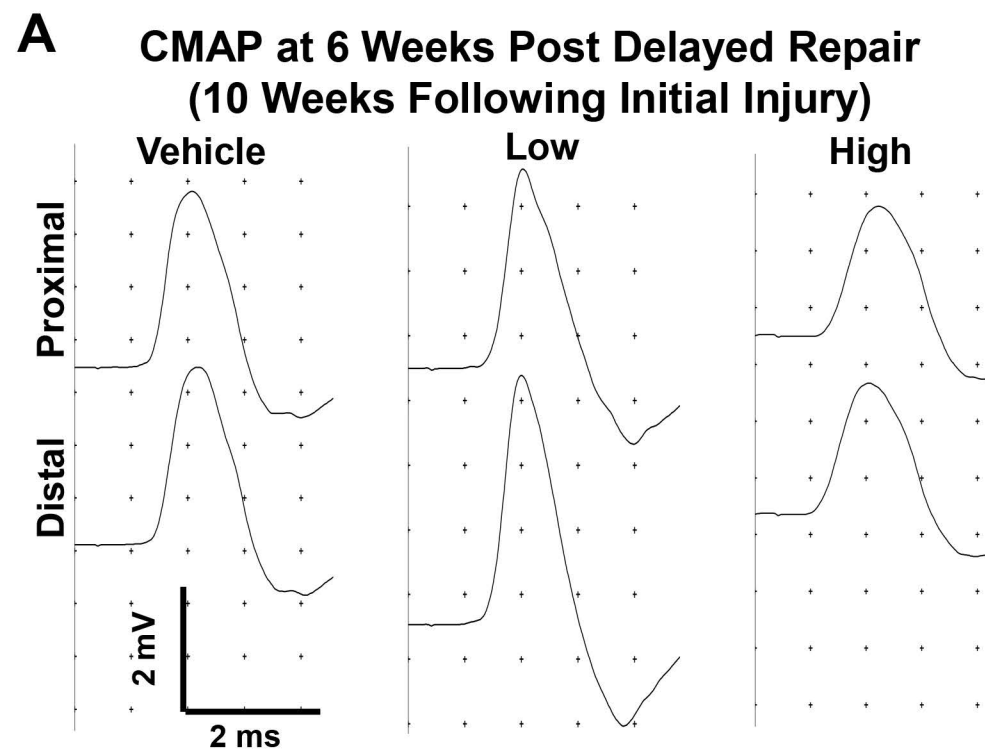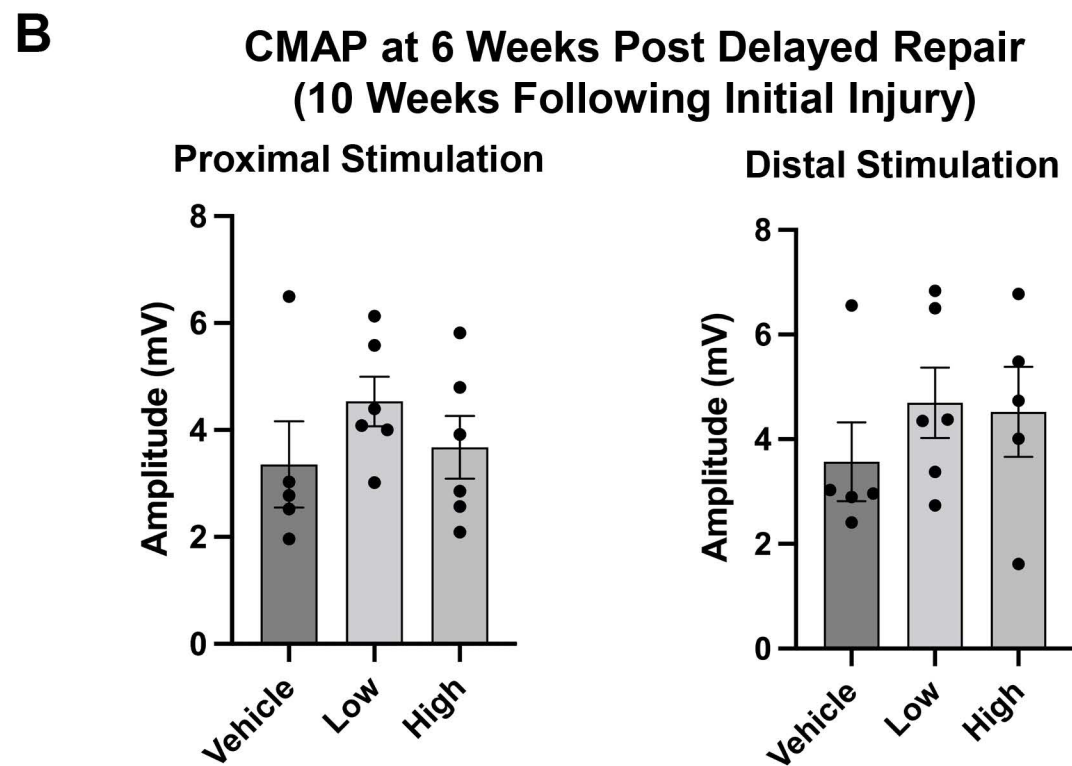

Supplement: Supplementary file 4 [file Image_4.pdf]

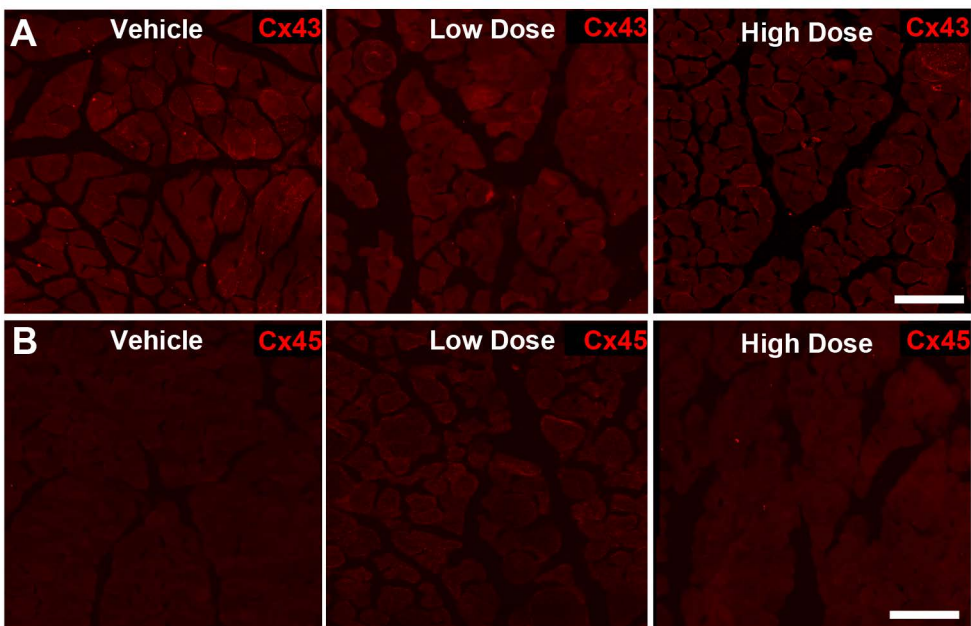

**C** Connexin 43 Expression

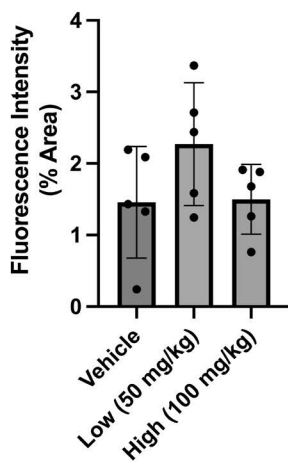

**D** Connexin 45 Expression

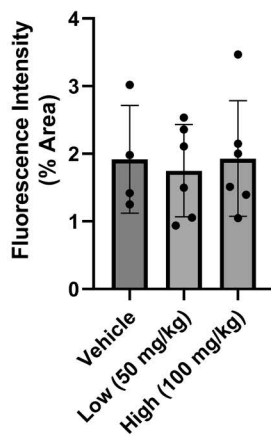

Supplement: Supplementary file 5 [file Image_5.pdf]
