# Supplementary material for: Orally administered boldine reduces muscle atrophy and promotes neuromuscular recovery in a rodent model of delayed nerve repair
Source: Front Cell Neurosci. 2023 Sep 27;17:1240916. doi: 10.3389/fncel.2023.1240916 (PMC10565860; doi:10.3389/fncel.2023.1240916)
Supplement: Supplementary file 3 [file Image_3.pdf]

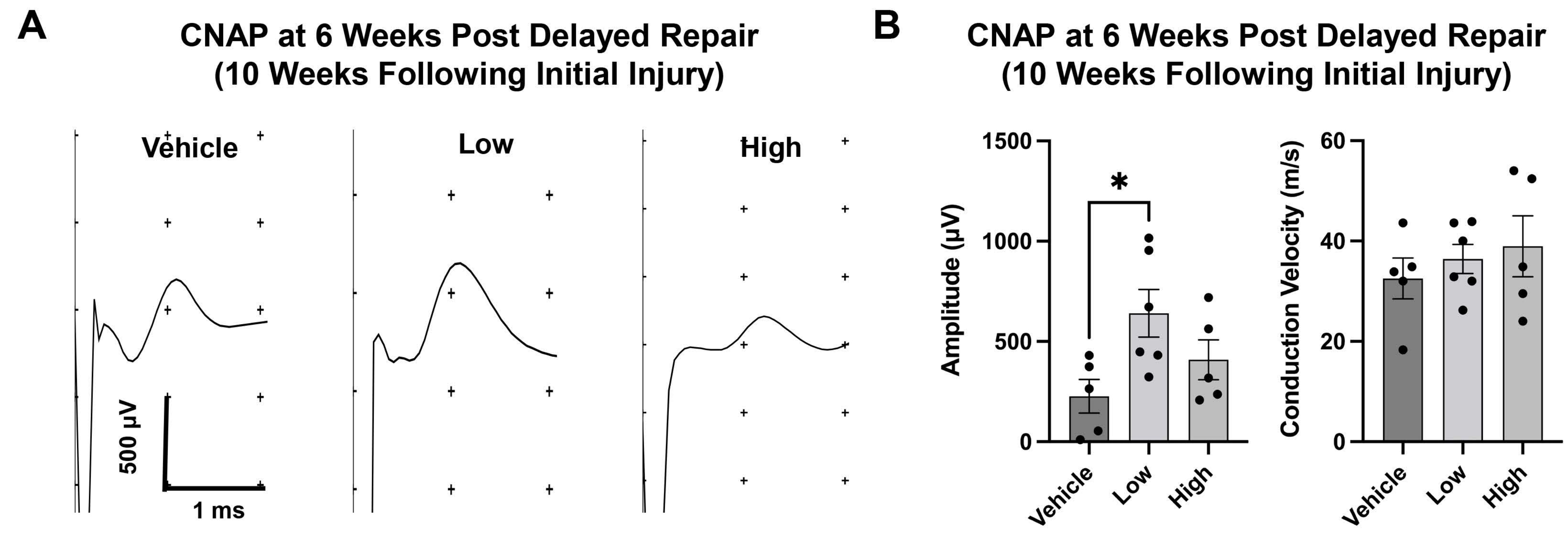
A
CNAP at 6 Weeks Post Delayed Repair
(10 Weeks Following Initial Injury)
Vehicle
Low
High
500 µV
1 ms
B
CNAP at 6 Weeks Post Delayed Repair
(10 Weeks Following Initial Injury)
Amplitude (µV)
1500
1000
500
0
*
Vehicle
Low
High
Conduction Velocity (m/s)
60
40
20
0
Vehicle
Low
High
